# Identification of Hepatocellular Carcinoma Subtypes Based on Global Gene Expression Profiling to Predict the Prognosis and Potential Therapeutic Drugs

**DOI:** 10.3390/biomedicines13010236

**Published:** 2025-01-20

**Authors:** Cunzhen Zhang, Jiyao Wang, Lin Jia, Qiang Wen, Na Gao, Hailing Qiao

**Affiliations:** Institute of Clinical Pharmacology, School of Basic Medical Sciences, Zhengzhou University, Zhengzhou 450001, China

**Keywords:** hepatocellular carcinoma, subtypes, diagnostic model, immune microenvironment, drug sensitivity

## Abstract

**Background:** Hepatocellular carcinoma (HCC) is a highly heterogeneous tumor, and distinguishing its subtypes holds significant value for diagnosis, treatment, and the prognosis. **Methods:** Unsupervised clustering analysis was conducted to classify HCC subtypes. Subtype signature genes were identified using LASSO, SVM, and logistic regression. Survival-related genes were identified using Cox regression, and their expression and function were validated via qPCR and gene interference. GO, KEGG, GSVA, and GSEA were used to determine enriched signaling pathways. ESTIMATE and CIBERSORT were used to calculate the stromal score, tumor purity, and immune cell infiltration. TIDE was employed to predict the patient response to immunotherapy. Finally, drug sensitivity was analyzed using the oncoPredict algorithm. **Results:** Two HCC subtypes with different gene expression profiles were identified, where subtype S1 exhibited a significantly shorter survival time. A subtype scoring formula and a nomogram were constructed, both of which showed an excellent predictive performance. COL11A1 and ACTL8 were identified as survival-related genes among the signature genes, and the downregulation of COL11A1 could suppress the invasion and migration of HepG2 cells. Subtype S1 was characterized by the upregulation of pathways related to collagen and the extracellular matrix, as well as downregulation associated with the xenobiotic metabolic process and fatty acid degradation. Subtype S1 showed higher stromal scores, immune scores, and ESTIMATE scores and infiltration of macrophages M0 and plasma cells, as well as lower tumor purity and infiltration of NK cells (resting/activated) and resting mast cells. Subtype S2 was more likely to benefit from immunotherapy. Subtype S1 appeared to be more sensitive to BMS-754807, JQ1, and Axitinib, while subtype S2 was more sensitive to SB505124, Pevonedistat, and Tamoxifen. **Conclusions:** HCC patients can be classified into two subtypes based on their gene expression profiles, which exhibit distinctions in terms of signaling pathways, the immune microenvironment, and drug sensitivity.

## 1. Introduction

Hepatocellular carcinoma (HCC) is the second-leading cause of cancer-related deaths worldwide, with known risk factors including viral infections, excessive alcohol consumption, and aflatoxin exposure [1,2]. Emerging evidence has highlighted the critical role of the tumor microenvironment in the initiation, progression, and prognosis of HCC, making it a promising target for novel cancer therapies [3,4,5,6]. For instance, diacylglycerol kinase gamma (DGKG) in tumor endothelial cells (ECs) has been shown to promote HCC progression, suggesting its potential as a therapeutic target [7]. The inhibition of 3-ketoacyl-CoA thiolase 1 (OXCT1) in tumor-associated macrophages (TAMs) has been proposed as a promising strategy for HCC treatment [6]. Additionally, the CNEPO protein has been linked to the infiltration of regulatory T cells (Tregs) in the tumor microenvironment and promotes HCC progression, suggesting that targeting its function may be beneficial for HCC patients [8]. Despite these promising findings, only Sorafenib and Regorafenib have been approved for the treatment of advanced HCC to date [9]. Therefore, there is an urgent need for further research into the underlying mechanisms of HCC and the development of novel therapeutic agents.

Over the past decade, significant advancements have been made in personalized medicine, enabling the development of tailored treatment plans for patients with diverse characteristics, thereby maximizing therapeutic efficacy and minimizing adverse effects [10]. The rapid development of bioinformatics and various omics sequencing technologies has greatly facilitated the advancement of personalized medicine [11,12,13,14]. Bioinformatics analysis allows for in-depth analysis of omics data and database information, enabling the identification of unique molecular signatures in individual patients, thus facilitating more precise diagnosis and the development of personalized treatment plans. For instance, the determination of gene mutations [15,16] and polymorphisms [17,18,19] in cancer patients has enabled personalized drug therapy in clinical practice.

The discovery of novel biomarkers and therapeutic targets has profound implications for the diagnosis and treatment of various diseases. Some biomarkers can serve both diagnostic and therapeutic purposes, offering significant advantages. For instance, MerTK has been shown to be a promising biomarker for predicting patient stratification with respect to HCC and a potential target to overcome resistance to anti-PD-1/PD-L1 therapy in HCC [20]. Studies have demonstrated that integrin receptors [21,22], HER2 [23], CLDN18 [24], GSH [25], and VEGF [26] can serve as personalized targets for tumors, and nanotechnology can be employed for targeted drug delivery to reduce drug toxicity and enhance therapeutic specificity and efficacy [27]. Moreover, some biomarkers can be used for tumor diagnosis and therapy through molecular imaging with radiolabeled tracers [28].

For this study, we leveraged publicly available transcriptomic data, coupled with bioinformatics analyses and experimental validation, in order to classify HCC subtypes and investigate the underlying mechanisms and drug sensitivities associated with different prognostic subtypes. Beyond the traditional focus on disease diagnosis, our study provides insights into the disease-related mechanisms and potential therapeutic strategies for various HCC subtypes, offering broader implications for pre-clinical and clinical researchers.

## 2. Materials and Methods

### 2.1. Data Collection and Processing

RNA-seq data of HCC and normal liver tissues were retrieved from The Cancer Genome Atlas (TCGA) database (https://cancergenome.nih.gov/ (accessed on 8 May 2024) and the NCBI Gene Expression Omnibus (GEO) database (https://www.ncbi.nlm.nih.gov/geo/ (accessed on 8 May 2024). From the TCGA database, RNA sequencing data and matching clinical information for 369 HCC patients and 110 normal liver tissues were acquired, and the data were transformed into the Log2 (TPM + 0.001) format). From the GEO database (GSE14520), RNA sequencing data and matching clinical information on 225 HCC patients and 220 normal tissues were collected.

### 2.2. Clustering Analysis

Clustering analysis of HCC samples was conducted using the R package “ConsensuClusterPlus” [29]. The optimal number of clusters was determined by selecting the k value that minimized the sum of squares within clusters, followed by 1000 repetitions to confirm the stability of classification. Kaplan–Meier (K-M) survival analysis was performed using SPSS, and survival curves were plotted with Prism. Principal component analysis (PCA) and differential gene analysis were conducted using the PCA and Limma functions in R. A cluster heatmap was employed to perform hierarchical clustering of the samples.

### 2.3. Identification of Subtype Signature Genes and Construction of Scoring Model

Least Absolute Shrinkage and Selection Operator (LASSO) regression and support vector machine (SVM) were used in R to screen the signature genes. Univariate and multivariate logistic regression were performed using the SPSS software to identify statistically significant (*p* < 0.05) subtype signature genes. A multivariate logistic regression model was then utilized to obtain regression coefficients of the subtype signature genes in order to construct a subtype scoring formula. Nomograms were built using functions such as Survival, RMS, and nomogramFormula in R. The model’s reliability was assessed through calibration and decision curve analysis (DCA) curves. Univariate and multivariate Cox regression analyses were performed to identify survival-related genes among the subtype signature genes. Protein–protein interaction (PPI) analysis was conducted using STRING (http://string-db.org (accessed on 6 August 2024).

### 2.4. Enrichment of Signaling Pathways

Differentially expressed genes (DEGs) between subtypes were identified using the Limma package in R [30], with screening criteria at |log FC| > 1 and adjusted *p* < 0.05. The R package “clusterProfiler” was utilized for Gene Ontology (GO) and Kyoto Encyclopedia of Genes and Genomes (KEGG) analyses [31]. Gene set variation analysis (GSVA) and gene set enrichment analysis (GSEA) were performed using the GSVA and GSEA packages in R to analyze differential pathways and enriched pathways between subtypes.

### 2.5. Immune Cell Infiltration

The R package “ESTIMATE” was used to analyze the stromal score, immune score, ESTIMATE score, and tumor purity of the samples [32]. The immune cell infiltration scores of different samples were determined using CIBERSORT [33].

### 2.6. Prediction of Immunotherapy Response

TPM data from TCGA-LIHC were normalized via mean sample expression, following which immune response prediction was performed using TIDE (http://tide.dfci.harvard.edu/ (accessed on 22 August 2024)).

### 2.7. Drug Sensitivity

Drug sensitivity analysis was performed using the R package oncoPredict [34,35], based on the GDSC database.

### 2.8. Collection of Tumor and Normal Tissues

A total of 11 liver tissue samples were collected from patients undergoing hepatic surgery at the First Affiliated Hospital of Zhengzhou University. These samples were divided into two groups: normal liver with non-HCC diseases (n = 5) and tumor tissues from patients with HCC (n = 6). This study was conducted in accordance with the declaration of Helsinki, and the protocol was approved by the ethics committee of Zhengzhou University (ZZUIRB2022-152) on 21 March 2022. Participants provided written informed consent.

### 2.9. RT-qPCR

Total RNA was extracted using TRIzol reagent (Vazyme, Nanjing, China). Reverse transcription was conducted using HiScript II Q RT SuperMix (Vazyme, Nanjing, China). Gene expression levels were measured via qPCR using Taq Pro Universal SYBR qPCR Master Mix (Vazyme, Nanjing, China) with GAPDH as an internal control. Expression levels were calculated using the 2^−ΔΔ^CT method.

### 2.10. Cell Culture and Infection

HepG2 cells, purchased from the National Collection of Authenticated Cell Cultures, were maintained in DMEM (ThermoFisher, Waltham, MA, USA) containing 10% FBS (Cyagen, Suzhou, China) at 37 °C with 5% CO_2_. A small interfering RNA (siRNA) guide (5′-AUACUCAUAGUCAUAUUCGAU-3′), passenger (5′-CGAAUAUGACUAUGAGUAUGG-3′), and negative control (5′-UUCUCCGAACGUGUCACGUTT-3′) were selected to reduce COL11A1 expression in HepG2 cells using lipofectamine 3000 transfection reagent (Invitrogen, Waltham, MA, USA), according to the manufacturer’s instructions.

### 2.11. Cell Invasion and Migration

For the scratch assay, cells were seeded in 6-well plates (Corning, Corning, NY, USA) and cultured in serum-free medium to 80% confluence. After creating a scratch, cells were cultured in 10% FBS-DMEM for 24 h. Cell debris was removed by washing with PBS. The scratch width was measured from photographs taken at 0 and 48 h. The migration rate was calculated as follows:

Migration% = (Scratch width_-0h_ − Scratch width_-48h_)/Scratch width_-0h_ (1)

For the invasion assay, trans-well filters were coated with Matrigel and seeded with 1 × 10^4^ cells/mL in 200 μL serum-free DMEM. The lower well contained 500 μL DMEM with 10% FBS. After incubation at 37 °C for 48 h, cells attached to the underside of the filter were stained with crystal violet and counted under a microscope at 200× magnification.

### 2.12. Statistical Analysis

Statistical analyses were performed using R (version 4.3.2) and SPSS (version 27.0). Differences between two groups for continuous variables were compared using Student’s *t*-test (for normal distribution) or the Wilcoxon test (for non-normal distribution). Categorical variables were compared using the Chi-square test. Univariate and multivariate logistic and Cox regression analyses were conducted using SPSS. Receiver operating characteristic (ROC) analysis was used to determine the optimal cutoff values for the predictor. Correlation analysis was performed using Spearman’s correlation. The Benjamini–Hochberg method was used to adjust *p*-values. *p* < 0.05 was considered statistically significant.

## 3. Result

### 3.1. Identification of HCC Patient Subtypes

**Figure 1:**
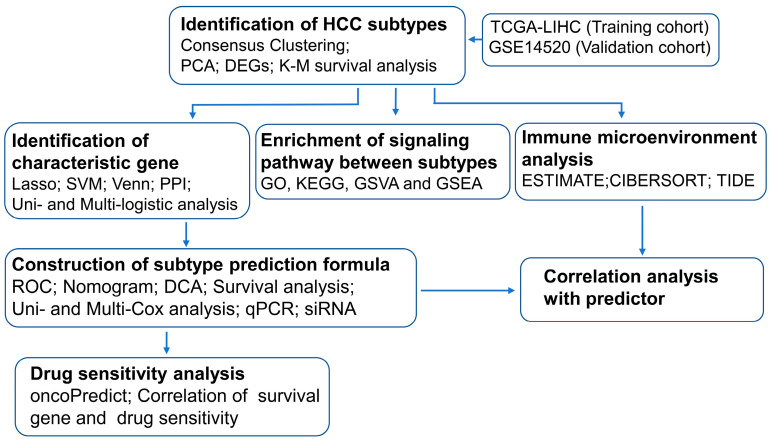
Flow chart of this study.

**Figure 2:**
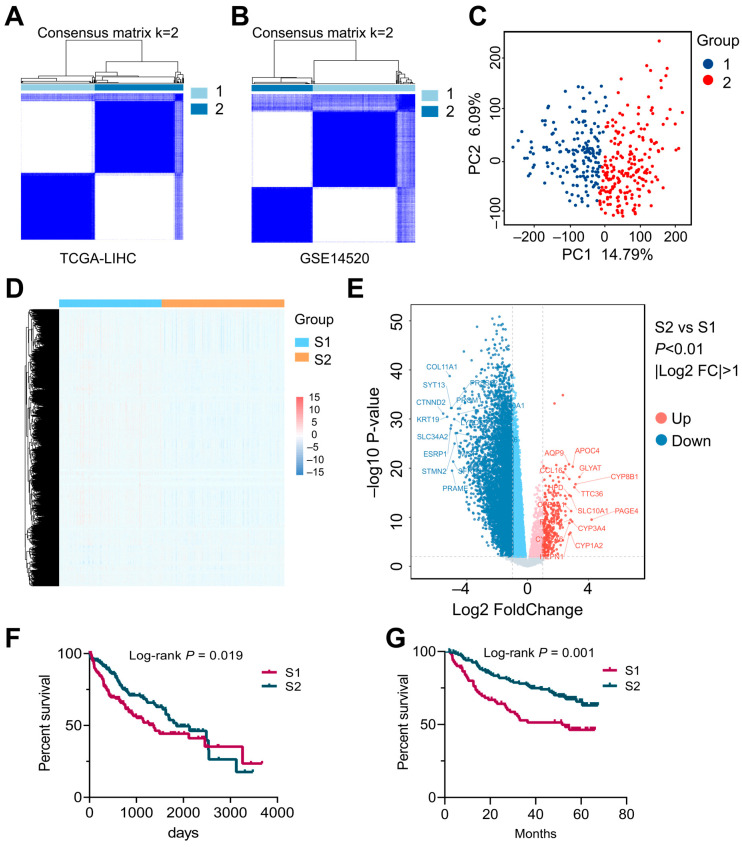
Identification of HCC subtypes. (**A**) Heatmap of the consensus matrix for two clusters in TCGA-LIHC (k = 2). (**B**) Heatmap of the consensus matrix for two clusters in GSE14520 (k = 2). (**C**) Principal component analysis (PCA) of two subtypes. (**D**) Clustering heatmap of S1 and S2 subtypes. (**E**) Differentially expressed genes between S1 and S2 subtypes. (**F**,**G**) K-M survival analysis of S1 and S2 subtypes based on OS (log-rank test) in the TCGA training cohort (**F**) and GSE14520 validation cohort (**G**). *p*-values were determined using Student’s *t*-test and the Wilcoxon test.

The flowchart of this study is exhibited in Figure 1. To determine whether different subtypes could be identified based on the overall gene expression profiles of hepatocellular carcinoma (HCC) patients, unsupervised clustering analysis was performed. The results indicated that, in the TCGA-LIHC dataset (training set), the highest intra-group consistency was achieved when K = 2, effectively dividing samples into two subgroups (Figure 2A, Supplementary Figure S1A,B), and the results for the GSE14520 dataset (validation set) were similar (Figure 2B, Supplementary Figure S1C,D). Principal component (PCA), cluster heatmap, and differentially expressed gene (DEG) analyses demonstrated significant differences between the two subtypes (Figure 2C–E). Kaplan–Meier (K-M) survival analysis revealed significant differences in median overall survival (OS) times between the two HCC patient subtypes in both the training and validation sets (Figure 2F, *p* = 0.019; Figure 2G, *p* = 0.001). Specifically, the median OS time for subtype 1 (S1) was notably shorter than that for subtype 2 (S2). In conclusion, these results suggest that HCC patients can be classified into two subtypes, based on their overall gene expression profiles with distinct OS times.

### 3.2. Construction of the Subtype Prediction System

**Figure 3:**
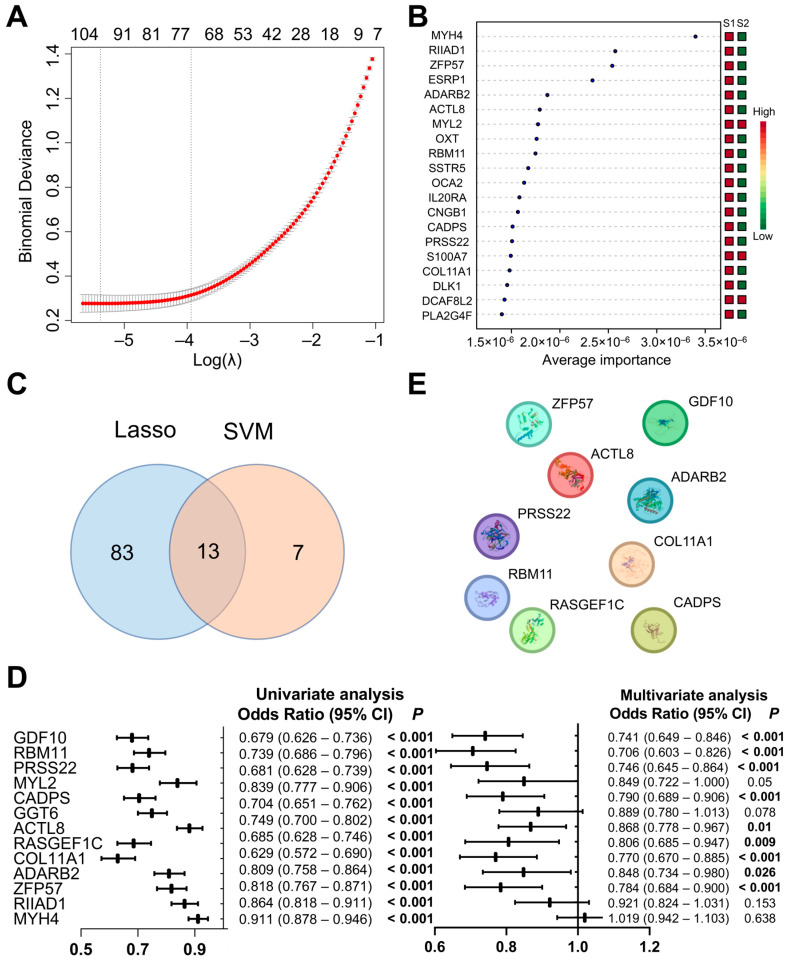
Identification of characteristic genes. (**A**,**B**) Screening of characteristic genes in S1 and S2 subtypes using LASSO regression (**A**) and SVM (**B**). (**C**) Cross-validation of LASSO regression and SVM. (**D**) Univariate and multivariate logistic regression in S1 and S2 subtypes. (**E**) Protein–protein interaction (PPI) analysis of characteristic genes.

To differentiate the two HCC subtypes, we identified characteristic genes using Least Absolute Shrinkage and Selection Operator (LASSO) regression and the machine learning algorithm support vector machine (SVM). LASSO regression identified 96 signature genes (Figure 3A, Supplementary Table S1). To simplify the classification model, intersection with the top 20 genes from the SVM analysis was conducted, resulting in 13 signature genes (Figure 3B,C). Univariate logistic regression analysis indicated that all 13 genes were independent factors affecting the classification. Multivariate logistic regression analysis identified nine genes as common factors influencing the classification (Figure 3D). The prediction formula for identifying patient subtype was as follows: Predictor = (−0.25 × ZFP57) + (−0.17 × ADARB2) + (−0.29 × COL11A1) + (−0.22 × RASGEF1C) + (−0.15 × ACTL8) + (−0.22 × CADPS) + (−0.32 × PRSS22) + (−0.36 × RBM11) + (−0.31 × GDF10) − 12.06. Protein–protein interaction (PPI) analysis showed no interactions among the nine signature genes, reinforcing their representativeness as a predictor for HCC subtypes (Figure 3E).

### 3.3. Evaluation of the Subtype Scoring System

**Figure 4:**
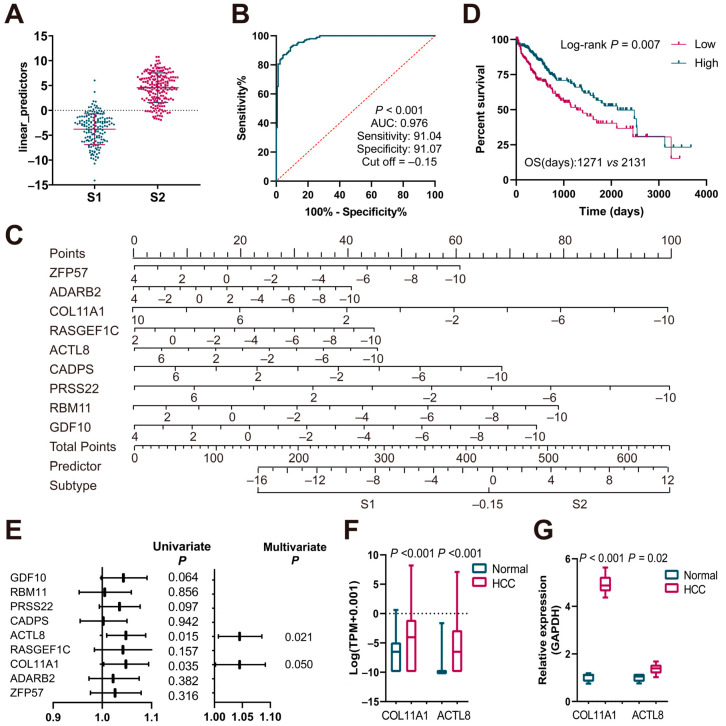
Construction and evaluation of the subtype prediction formula. (**A**) Distribution of predictor in S1 and S2 subtypes. (**B**) ROC analysis of predictor. (**C**) Nomogram for the identification of S1 and S2 subtypes. (**D**) K-M survival analysis between low and high predictor groups based on OS (log-rank test). (**E**) Univariate and multivariate Cox regressions on the characteristic genes. (**F**) Expression of COL11A1 and ACTL8 in the TCGA cohort. (**G**) Expression of COL11A1 and ACTL8 between normal and HCC patients (normal n = 5, HCC n = 6).

To assess whether the predictor could distinguish between the two subtypes of patients, the predictor distribution among the subtypes was examined, revealing minimal overlap in only a few patients (Figure 4A). Receiver operating characteristic (ROC) analysis illustrated that the predictor could effectively distinguish between the two subtypes, with an AUC of 0.976. At the optimal cutoff value of −0.154, both sensitivity and specificity were greater than 0.91 (Figure 4B). For clinical convenience in distinguishing patient subtypes, a nomogram was constructed (Figure 4C) and its predictive capability was assessed. In the calibration curve, the prediction curve almost completely overlapped with the diagonal line, indicating a good predictive performance and high consistency between the predicted probabilities and actual results (Supplementary Figure S2A). Decision curve analysis (DCA) curves also demonstrated the good predictive performance of the model (Supplementary Figure S2B). Patients were categorized based on the predictor value of -0.154, revealing that those with lower predictor values had significantly shorter median OS times (*p* = 0.007) (Figure 4D).

To identify survival-associated genes among the nine signature genes, univariate and multivariate Cox analyses were conducted. Both COL11A1 (collagen type XI alpha 1) and ACTL8 (actin-like protein 8) emerged as significant factors influencing patient survival in the univariate and multivariate analyses (Figure 4E). TCGA-LIHC (Figure 4F) and qPCR analyses on human liver tissues (Figure 4G) confirmed that these two genes were elevated in tumor tissues compared to normal tissues.

### 3.4. Effect of COL11A1 Downregulation on the Invasion and Migration of HepG2 Cells

**Figure 5:**
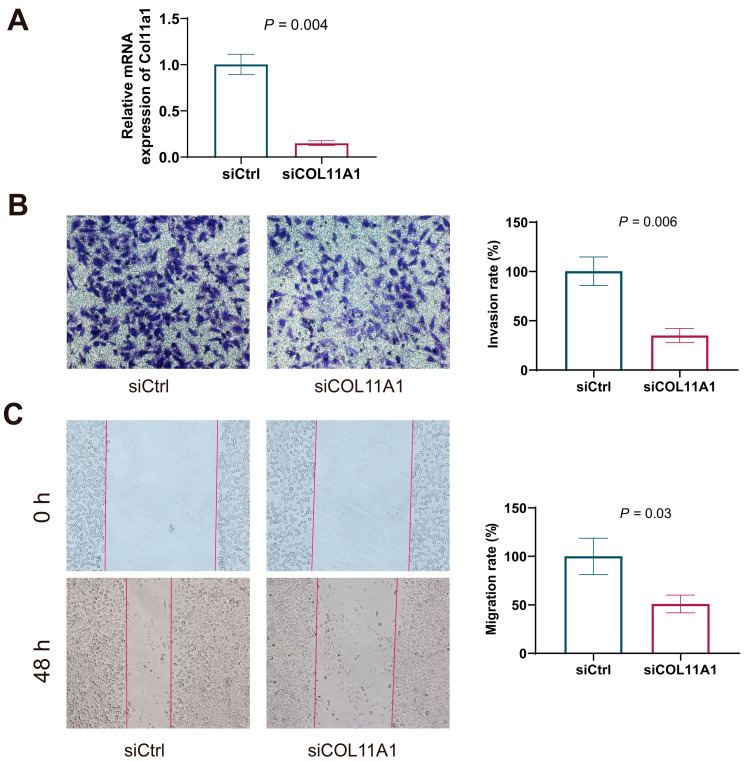
Downregulated Col11a1 inhibited the invasion and migration of HepG2 cells. (**A**) Col11a1 mRNA expression was significantly downregulated in HepG2 cells by col11a1 siRNA (*t*-test). (**B**) Invasion of HepG2 cells detected using the trans-well assay (*t*-test). (**C**) Migration of HepG2 detected using the scratch assay (*t*-test).

Given the significantly higher expression of COL11A1 in tumor tissues, we further explored its influence on cell invasion and migration. COL11A1 siRNA transfection significantly reduced the mRNA expression of Col11a1 in HepG2 cells (Figure 5A). Trans-well assays showed that cell invasion was significantly inhibited in HepG2 cells transfected with Col11a1 siRNA (Figure 5B). Additionally, scratch assays demonstrated that downregulating COL11A1 expression significantly inhibited cell migration in HepG2 cells (Figure 5C). Taken together, these results suggest that COL11A1 is associated with tumor cell invasion and migration and, thus, inhibiting its expression can suppress invasion and migration in HepG2 cells.

### 3.5. Differential Signaling Pathways Between Two HCC Subtypes

**Figure 6:**
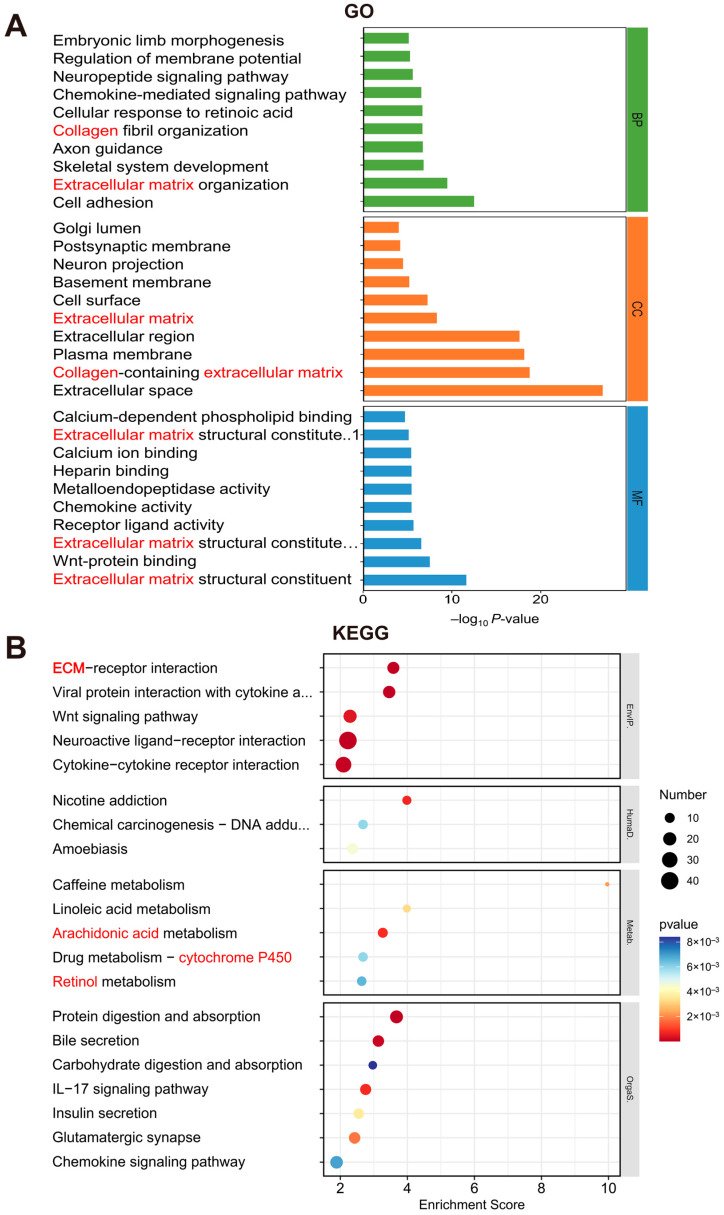
Enrichment analysis of signaling pathways in S1 and S2 subtypes. (**A**) Gene Ontology (GO) analysis of DEGs between two subtypes. (**B**) Kyoto Encyclopedia of Genes and Genomes (KEGG) analysis of DEGs between two subtypes.

To investigate the differential pathways between the two subtypes, an enrichment analysis was conducted. Gene Ontology (GO) analysis revealed that DEGs were primarily involved in pathways related to cell adhesion, extracellular matrix organization, collagen fibril organization, the cellular response to retinoic acid, the chemokine-mediated signaling pathway, and the collagen-containing extracellular matrix, with a significant involvement in extracellular-matrix-related pathways (Figure 6A). Kyoto Encyclopedia of Genes and Genomes (KEGG) analysis indicated that DEGs significantly participated in ECM–receptor interaction, cytokine–cytokine receptor interaction, arachidonic acid metabolism, CYP450 metabolism, retinol metabolism, bile secretion, and the chemokine signaling pathway (Figure 6B).

**Figure 7:**
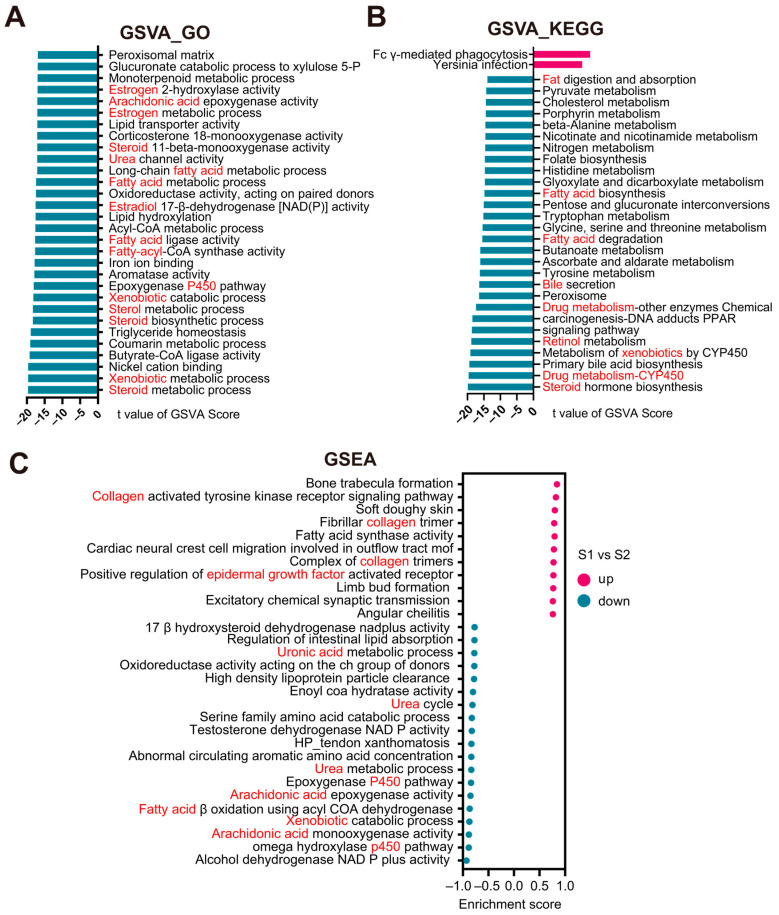
Enrichment analysis of signaling pathways in S1 and S2 subtypes. (**A**) Gene set variation analysis (GSVA)-GO between two subtypes. (**B**) GSVA-KEGG between two subtypes. (**C**) Gene set enrichment analysis (GSEA) between two subtypes.

Gene set variation analysis (GSVA)–GO enrichment analysis showed that downregulated pathways in subtype S1 primarily involved the steroid metabolic process, xenobiotic metabolic process, CYP450 pathway, fatty acid metabolic process, and arachidonic acid epoxygenase activity (Figure 7A). GSVA-KEGG enrichment analysis demonstrated that downregulated pathways in subtype S1 were mainly related to steroid hormone biosynthesis, drug metabolism by CYP450, bile acid biosynthesis and secretion, metabolism of xenobiotics by CYP450, fatty acid degradation and biosynthesis, and cholesterol metabolism (Figure 7B).

The gene set enrichment analysis (GSEA) results revealed that the most significantly upregulated pathways in subtype S1 included the collagen-activated tyrosine kinase receptor signaling pathway, fatty acid synthase activity, and positive regulation of epidermal growth factor receptor activity (Figure 7C). Downregulated pathways predominantly consisted of the omega hydroxylase P450 pathway, arachidonic acid monooxygenase activity, xenobiotic catabolic process, and fatty acid β-oxidation using acyl-CoA dehydrogenase (Figure 7C).

In summary, the upregulated pathways in subtype S1 mainly involve collagen and the extracellular matrix, while the downregulated pathways primarily include xenobiotic metabolism, fatty acids, arachidonic acid, steroids, retinol, retinoic acid, CYP450, bile secretion, and urea-related.

### 3.6. Immune Microenvironment in Two HCC Subtypes

**Figure 8:**
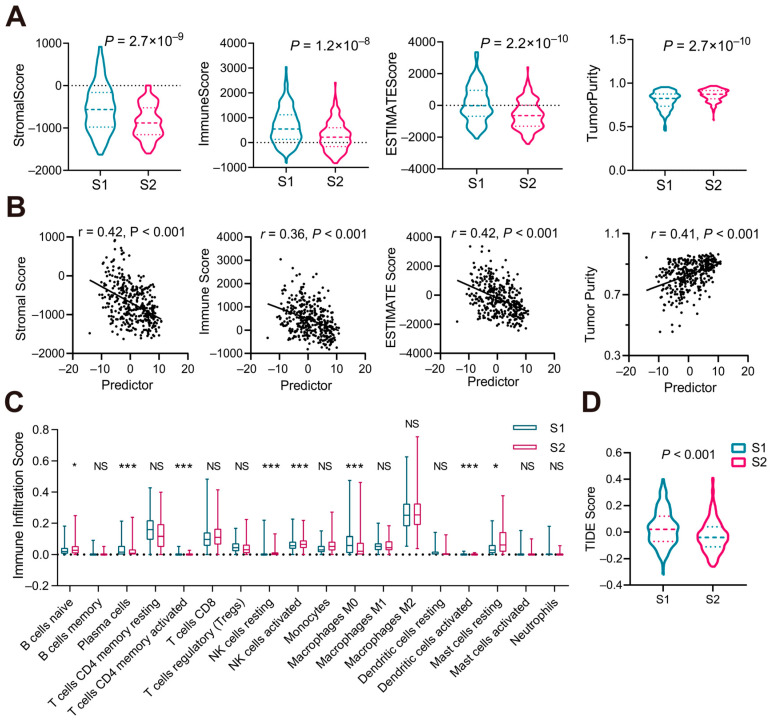
Immune microenvironment characteristics in S1 and S2 subtypes. (**A**,**B**) Stromal score, immune score, ESTIMATE score, and tumor purity (**A**) and their correlation with the subtype predictor (**B**). (**C**) Immune cell infiltration characteristics of two subgroups. (**D**) TIDE scores of S1 and S2 subtypes. *p*-values were determined via Student’s *t*-test and the Wilcoxon test; correlation analysis was performed via Spearman analysis.

To examine the differences in immune microenvironments between the two subtypes, ESTIMATE, CIBERSORT, and Tumor Immune Dysfunction and Exclusion (TIDE) analyses were conducted. The ESTIMATE results revealed that subtype S1 exhibited higher stromal scores, immune scores, and ESTIMATE scores and lower tumor purity (Figure 8A). Correlation analysis indicated that the predictor was negatively correlated with stromal scores, immune scores, and ESTIMATE scores, while it was positively correlated with tumor purity (Figure 8B).

CIBERSORT analysis showed that subtype S1 had higher infiltration levels of macrophages M0, plasma cells, activated memory CD4 T cells, and activated dendritic cells, while it had lower infiltration levels of resting NK cells, activated NK cells, resting mast cells, and naïve B cells (Figure 8C). There was a positive correlation between the predictor and the infiltration levels of resting mast cells, monocytes, and resting NK cells, while a negative correlation was observed between the predictor and the infiltration levels of macrophages M0, resting dendritic cells, resting memory CD4 T cells, neutrophils, regulatory T cells, plasma cells, and memory B cells (Table 1, Supplementary Figure S3).

Next, we evaluated the difference in the clinical response to immunotherapy between the two subtypes using the Tumor Immune Dysfunction and Exclusion (TIDE) score. The results indicated that subtype S1 had higher TIDE scores, suggesting that subtype S1 was more likely to evade immune responses and had limited benefit from immunotherapy, while subtype S2 might benefit more (Figure 8D).

Overall, these results demonstrate significant differences in stromal scores, immune scores, ESTIMATE scores, tumor purity, immune cell infiltration levels, and TIDE scores between the two subtypes, which may be crucial factors contributing to the differences in patient prognosis.

### 3.7. Drug Sensitivity Analysis

To explore potential therapeutic differences between the two HCC subtypes, drug sensitivity analysis was performed. The results indicate that subtype S1 may be more sensitive to BMS.754807, JQ1, Axitinib, KU.55933, Tozasertib, PF.4708671, Navitoclax, ABT737, and Ibrutinib (Table 2, Supplementary Figure S4A–I), while subtype S2 may be more sensitive to SB505124, Pevonedistat, Tamoxifen, AT13148, Rapamycin, BMS.536924, BMS.345541, Dactolisib, and Sorafenib (Table 2, Supplementary Figure S5A–I). Based on our previous results demonstrating that inhibiting COL11A1 expression could suppress invasion and migration in HepG2 cells, the correlation between COL11A1 expression and sensitivity to subtype S1-sensitive drugs was analyzed. Our results revealed a significant correlation (Table 2, Supplementary Figure S6A–I), suggesting that these drugs may represent a promising therapeutic strategy for HCC, particularly for tumors with the S1 subtype.

## 4. Discussion

The results of this study revealed that HCC patients can be classified into two subtypes, based on their gene expression profiles, using unsupervised clustering analysis. This classification is consistent with other studies that have identified two subtypes of HCC patients (proliferation and non-proliferation) [36,37,38,39]. Survival analysis indicated significant prognostic differences between the two subtypes, suggesting distinct mechanisms of disease progression—and, therefore, different therapeutic strategies—for each subtype. Drug sensitivity analysis further demonstrated differences in drug responsiveness between the subtypes.

K-M survival analysis of the HCC subtype-specific genes identified COL11A1 and ACTL8 as both signature and survival-related genes. COL11A1 is a fibrillar collagen prevalent in the basement membrane, which is a major component of the extracellular matrix. It is significantly upregulated in various types of tumors. Studies have shown that COL11A1 promotes tumor cell invasion through multiple mechanisms, leading to a poor prognosis in diseases such as ovarian cancer, head and neck squamous cell carcinoma [40,41], pancreatic cancer [42], gastric cancer [43], colorectal cancer [44], and certain sarcomas [45]. Additionally, COL11A1 is associated with breast cancer metastasis and has been used as a diagnostic marker to distinguish between invasive and non-invasive breast cancer [46,47,48]. ACTL8 is a member of the cancer/testis antigens (CTA) family. Research has indicated that ACTL8 is linked to a poor prognosis in glioblastoma and breast cancer [49,50]. Positive associations between ACTL8 and the progression of colorectal cancer (CRC) [51], head and neck squamous cell carcinoma (HNSCC) [49], and non-small-cell lung cancer (NSCLC) [52] have also been reported. Several studies have suggested that ACTL8 is immune-related and could be a potential target for tumor immunotherapy [53].

Various pathway enrichment analyses indicated that the upregulated pathways in subtype S1 (with a worse prognosis) primarily involve the extracellular matrix, collagen, and angiogenesis. In the analysis of survival-related genes, COL11A1 emerged as both a subtype-specific and survival-related gene, suggesting that the upregulation of collagen-related pathways might be a critical factor for the worse prognosis associated with subtype S1. ESTIMATE analysis revealed the relationships between the stromal score, immune score, ESTIMATE score, tumor purity, and the malignant progression of HCC, consistent with other types of tumors. For example, higher stromal scores were correlated with higher tumor stages in stomach adenocarcinoma patients [54]. In prostate cancer patients, low tumor purity was associated with shorter survival times and faster recurrence [55]. Traditional views hold that immune activation suppresses tumors, whereas immune suppression promotes them. Therefore, based on patient survival times, the predictor, and their relationships with immune cells, it is speculated that resting mast cells, monocytes, and resting NK cells may have immune-activating functions, while macrophages M0, resting dendritic cells, neutrophils, regulatory T cells, plasma cells, and memory B cells may have immune-suppressive roles. These findings suggest that collagen- and immune-related characteristics might play a vital role in determining the differences in survival between the two subtypes.

We selected sensitive drugs based on the inter-group significance (*p*-value) rather than the IC50. This is because some drugs with lower IC50 values may have been extensively studied, while drugs that are sensitive to specific subtypes may still demonstrate efficacy in those subtypes even if the overall IC50 is relatively high. We observed a strong negative correlation between the IC50 of sensitive drugs to the S1 subtype and COL11A1 expression, suggesting that the sensitivity of these drugs may be associated with COL11A1. In contrast, the IC50 of sensitive drugs to the S2 subtype showed a positive correlation with COL11A1 expression, indicating that the sensitivity of these drugs may be associated with other targets or mechanisms besides COL11A1.

While our study provides a foundation for elucidating the progression mechanisms of HCC and developing treatment strategies, some limitations must be acknowledged. First, the analysis was based on retrospective data from public datasets, which may introduce selection bias and limit the generalizability of our findings. Furthermore, our study was primarily conducted through the use of bioinformatics approaches, necessitating more experimental validation to confirm the results.

## 5. Conclusions

In summary, we developed a method to classify HCC patients into two subtypes based on their overall gene expression profiles, which show differences in enriched pathways, the immune microenvironment, and drug sensitivity. This provides a basis for developing more personalized HCC therapeutic strategies.

## Figures and Tables

**Table 1:** Correlation of immune cell infiltration and the subtype predictor.

Immune Cell	Correlation with Predictor(*r*)	*p*-Value
Mast cells resting	0.37	0.000
Monocytes	0.31	0.000
NK cells resting	0.24	0.000
T cells CD8	0.07	0.199
B cells naive	0.06	0.210
Macrophages M1	0.04	0.427
NK cells activated	0.04	0.497
T cells follicular helper	0.02	0.762
Macrophages M2	0.02	0.655
Plasma cells	−0.12	0.025
B cells memory	−0.12	0.031
T cells regulatory	−0.13	0.007
Neutrophils	−0.14	0.011
T cells CD4 memory resting	−0.18	0.001
Dendritic cells resting	−0.21	0.000
Macrophages M0	−0.25	0.000

**Table 2:** Drug sensitivity (IC50) and correlation with the expression of COL11A1.

Drug	IC50 Median (Quartile)		*p*-Value	Correlation with COL11A1 (*r*)	*p*-Value
	Subtype 1	Subtype 2			
BMS-754807_2171	0.15 (0.04–0.35)	2.56 (1.2–4.53)	2.90 × 10^−52^	−0.78	4.64 × 10^−75^
JQ1_2172	5.94 (4.19–8.28)	17.42 (12.07–24.05)	7.80 × 10^−46^	−0.68	3.85 × 10^−51^
Axitinib_1021	11.43 (8.63–14.71)	25.24 (18.89–34.76)	3.50 × 10^−43^	−0.69	1.73 × 10^−52^
KU-55933_1030	77.72 (72.06–83.68)	95.49 (88.61–102.31)	7.90 × 10^−40^	−0.76	1.84 × 10^−71^
Tozasertib_1096	12.17 (8.93–16.84)	25.38 (19.96–35.67)	3.20 × 10^−38^	−0.66	3.13 × 10^−47^
PF-4708671_1129	30.52 (25.04–41.5)	59.8 (46.95–76.3)	3.40 × 10^−38^	−0.58	1.29 × 10^−34^
Navitoclax_1011	3.77 (2.03–5.73)	11.24 (7.07–17.8)	1.30 × 10^−37^	−0.61	1.37 × 10^−38^
ABT737_1910	4.8 (2.31–7.34)	13.97 (8.93–21.27)	9.00 × 10^−36^	−0.63	2.37 × 10^−41^
Ibrutinib_1799	56.36 (36.25–80.62)	131.2 (90.93–204.32)	2.10 × 10^−34^	−0.58	4.17 × 10^−34^
SB505124_1194	11 (10.57–11.72)	9.54 (9.01–10.04)	2.40 × 10^−44^	0.76	3.42 × 10^−71^
Pevonedistat_1529	3.89 (2.42–6.98)	1.11 (0.69–1.64)	9.10 × 10^−41^	0.65	1.87 × 10^−45^
Tamoxifen_1199	45.62 (37.54–57.41)	28.32 (23.17–33.12)	3.30 × 10^−38^	0.55	1.74 × 10^−30^
AT13148_2170	60.19 (42.77–96.63)	25.19 (18.22–35.66)	1.10 × 10^−38^	0.61	2.19 × 10^−37^
Rapamycin_1084	0.18 (0.12–0.25)	0.08 (0.06–0.11)	3.30 × 10^−37^	0.57	1.09 × 10^−33^
BMS-536924_1091	12.14 (8.78–15.78)	6.04 (4.57–8)	8.00 × 10^−37^	0.46	1.09 × 10^−33^
BMS-345541_1249	43.99 (31.66–67.61)	19.16 (15.18–25.24)	9.00 × 10^−37^	0.65	1.09 × 10^−33^
Dactolisib_1057	0.28 (0.21–0.43)	0.14 (0.11–0.19)	2.10 × 10^−36^	0.48	1.09 × 10^−33^
Sorafenib_1085	19.89 (15.41–26.78)	10.82 (8.29–13.44)	1.10 × 10^−35^	0.67	6.75 × 10^−50^

## Data Availability

The datasets supporting the conclusions of this article are available in the TCGA-LIHC and GEO databases. https://portal.gdc.cancer.gov/ (accessed on 8 May 2024), https://www.genome.gov/ (accessed on 8 May 2024).

## Supplementary Materials

The following supporting information can be downloaded at https://www.mdpi.com/article/10.3390/biomedicines13010236/s1, Figure S1: Identification of HCC subtypes in TCGA-LIHC and GSE14520. (A) The consensus matrix’s CDF plot from k = 2–9 on TCGA-LIHC. (B) Relative change in the area under the CDF curve for k = 2 to 9 on TCGA-LIHC. (C) The consensus matrix’s CDF plot from k = 2–9 on GSE14520. (D) Relative change in the area under the CDF curve for k = 2 to 9 on GSE14520. Figure S2: The evaluation of predictive performance using scoring formulas. (A) Calibration curves for the prediction formula. (B) DCA curves of characteristic genes. Figure S3: Correlation of immune cell infiltration and subtype predictor. (A–P) are B cells naive, B cells memory, Plasma cells, T cells CD8, T cells CD4, T cells helper, Tregs, NK cells resting, NK cells activated, Monocytes, Macrophages M0, Macrophages M1, Macrophages M2, Dendritic cells resting, Mast cells resting, Neutrophils, respectively. Correlation analyses were performed via Spearman analysis. Figure S4: Sensitive drug in S1 subtype. (A–I) are BMS.754807, JQ1, Axitinib, KU.55933, Tozasertib, PF.4708671, Navitoclax, ABT737, and Ibrutinib, respectively. *p*-values were determined via Wilcoxon test. Figure S5: Sensitive drug in S2 subtype. (A–I) are SB505124, Pevonedistat, Tamoxifen, AT13148, Rapamycin, BMS.536924, BMS.345541, Dactolisib, and Sorafenib, respectively. *p*-values were determined via Wilcoxon test. Figure S6: Correlation between IC50 of sensitive drugs in S1 subtype and COL11A1. (A–I) are BMS.754807, JQ1, Axitinib, KU.55933, Tozasertib, PF.4708671, Navitoclax, ABT737, and Ibrutinib, respectively. Correlation analyses were performed via Spearman analysis. Table S1: Identification of characteristic genes via LASSO regression.

## Abbreviations

Hepatocellular carcinoma (HCC); principal component analysis (PCA); differentially expressed genes (DEGs); Kaplan–Meier (K-M); overall survival (OS); Least Absolute Shrinkage and Selection Operator (LASSO); support vector machine (SVM); receiver operating characteristic (ROC); decision curve analysis (DCA); collagen type XI alpha 1 (COL11A1); actin-like protein 8 (ACTL8); Gene Ontology (GO); Kyoto Encyclopedia of Genes and Genomes (KEGG); gene set variation analysis (GSVA); gene set enrichment analysis (GSEA); Tumor Immune Dysfunction and Exclusion (TIDE).
