# Supplementary material for: Identification of Hepatocellular Carcinoma Subtypes Based on Global Gene Expression Profiling to Predict the Prognosis and Potential Therapeutic Drugs
Source: Biomedicines. 2025 Jan 20;13(1):236. doi: 10.3390/biomedicines13010236 (PMC11761595; doi:10.3390/biomedicines13010236)
Supplement: Supplementary file 1 [file biomedicines-13-00236-s001.zip › biomedicines-3262765-supplementary.pdf]

biomedicines

MDPI

Supplementary

Identification of Hepatocellular Carcinoma Subtypes Based on Global Gene Expression Profiling to Predict the Prognosis and Potential Therapeutic Drugs

Cunzhen Zhang, Jiyao Wang, Lin Jia, Qiang Wen, Na Gao and Hailing Qiao *

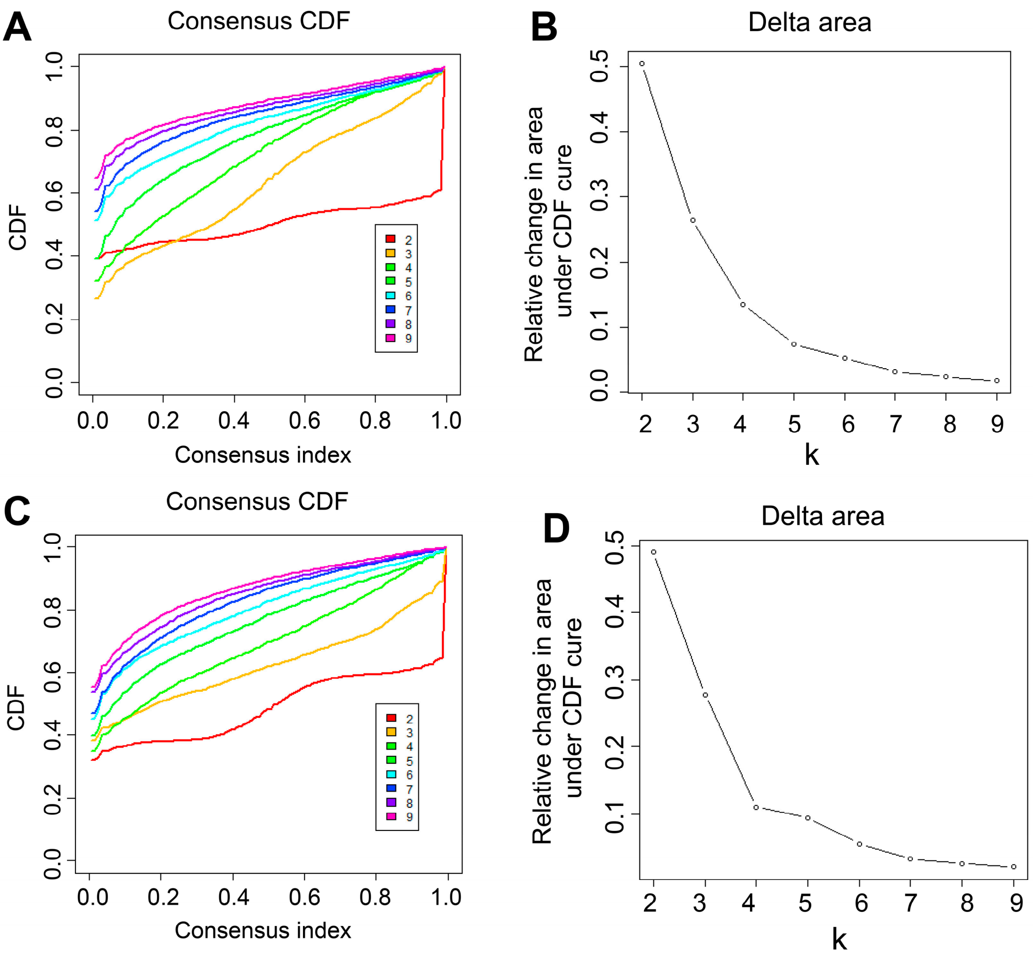

Figure S1. Identification of HCC subtypes in TCGA-LIHC and GSE14520. (**A**) The consensus matrix's CDF plot from k = 2–9 on TCGA-LIHC. (**B**) Relative change in the area under the CDF curve for k = 2 to 9 on TCGA-LIHC. (**C**) The consensus matrix's CDF plot from k = 2–9 on GSE14520. (**D**) Relative change in the area under the CDF curve for k = 2 to 9 on GSE14520.

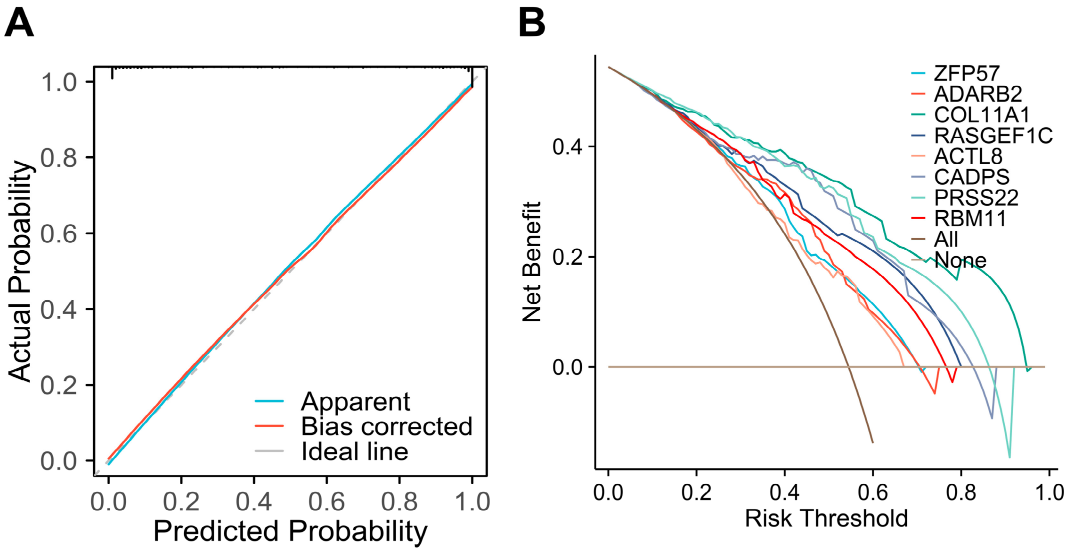

Figure S2. The evaluation of predictive performance using scoring formulas. (**A**) Calibration curves for the prediction formula. (**B**) DCA curves of characteristic genes.

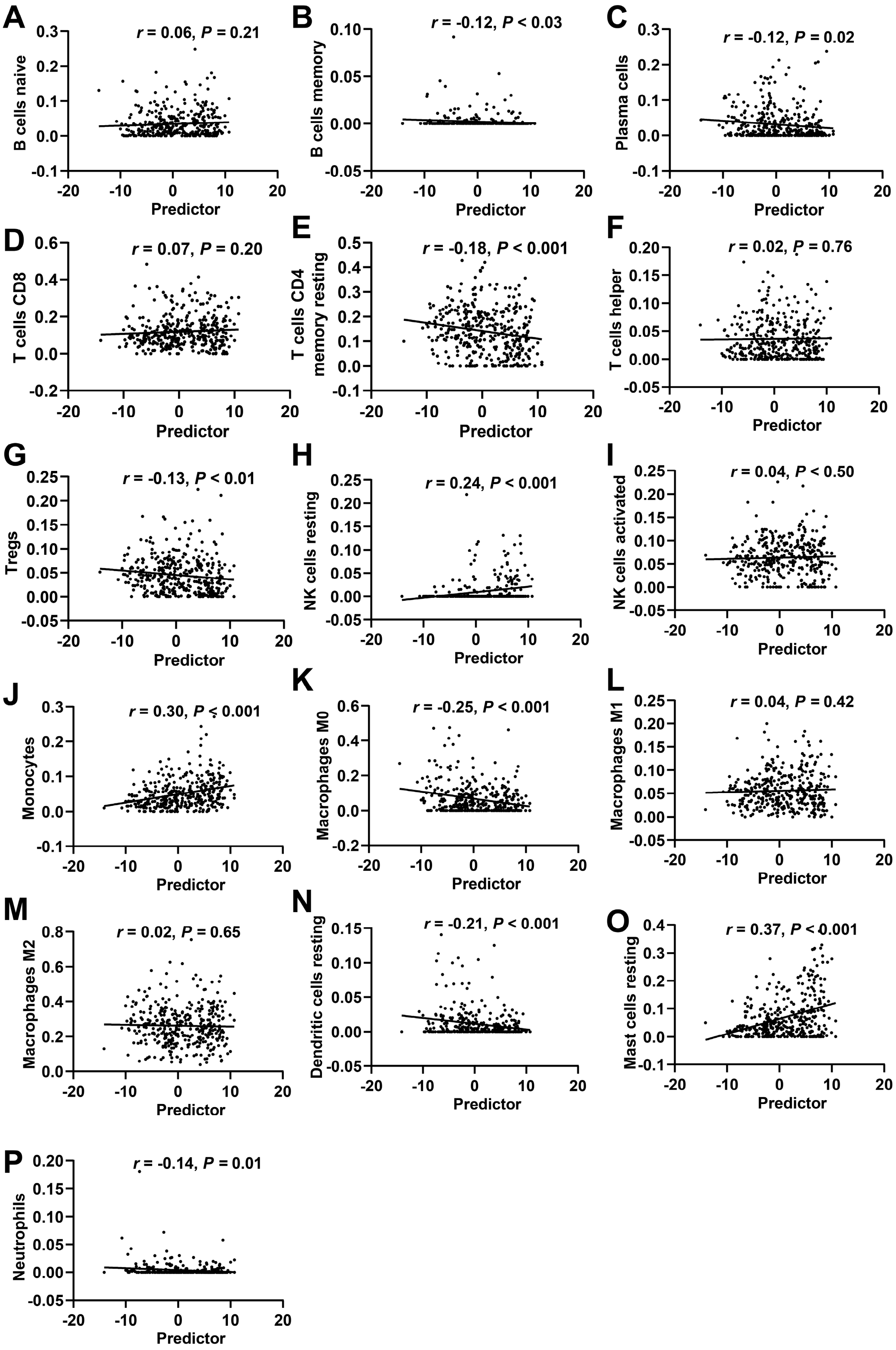

Figure S3. Correlation of immune cell infiltration and subtype predictor. (**A**–**P**) are B cells naive, B cells memory, Plasma cells, T cells CD8, T cells CD4, T cells helper, Tregs, NK cells resting, NK cells activated, Monocytes, Macrophages M0, Macrophages M1, Macrophages M2, Dendritic cells resting, Mast cells resting, Neutrophils, respectively. Correlation analyses were performed via Spearman analysis.

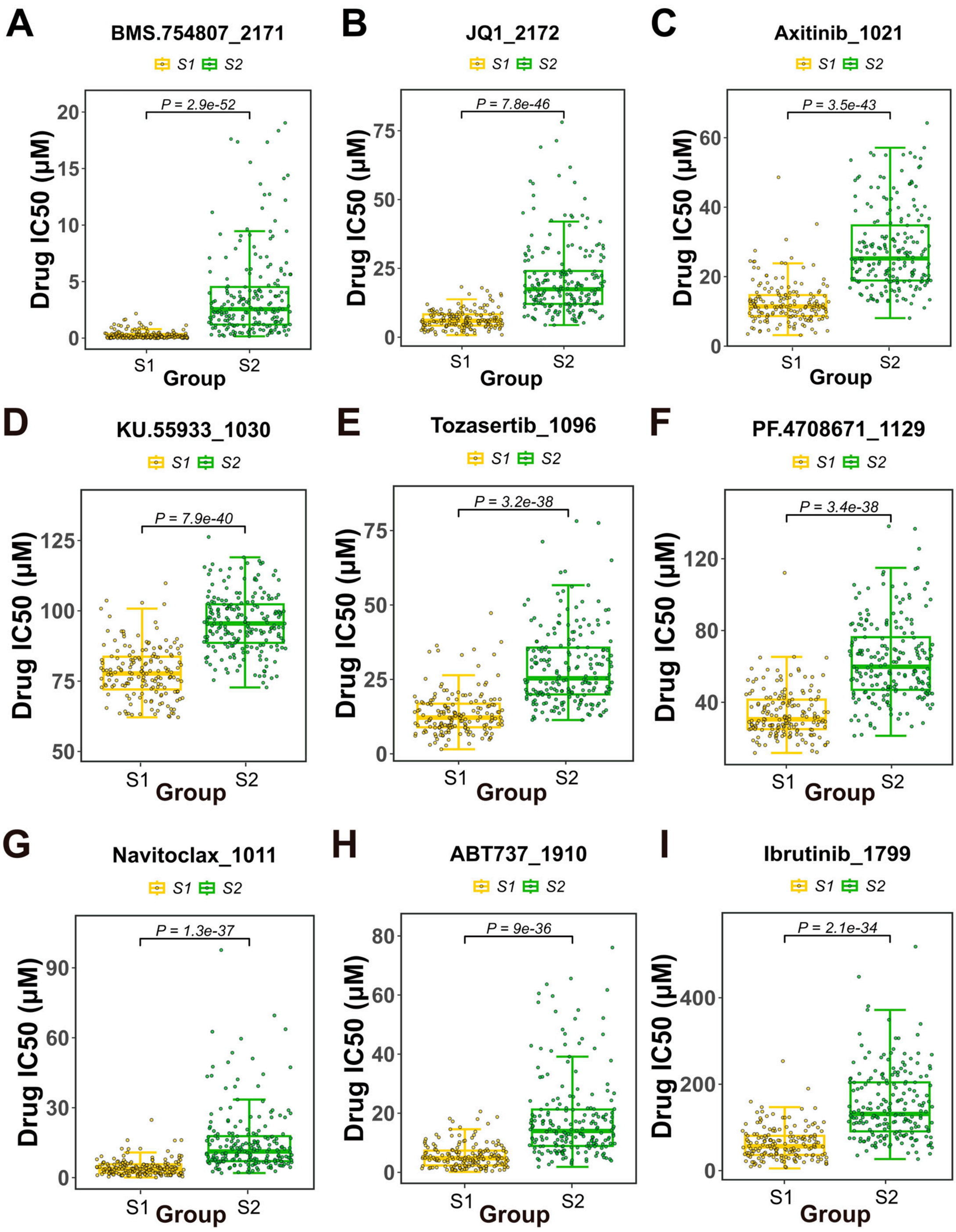

Figure S4. Sensitive drug in S1 subtype. (**A**–**I**) are BMS.754807, JQ1, Axitinib, KU.55933, Tozasertib, PF.4708671, Navitoclax, ABT737, and Ibrutinib, respectively. *p*-values were determined via Wilcoxon test.

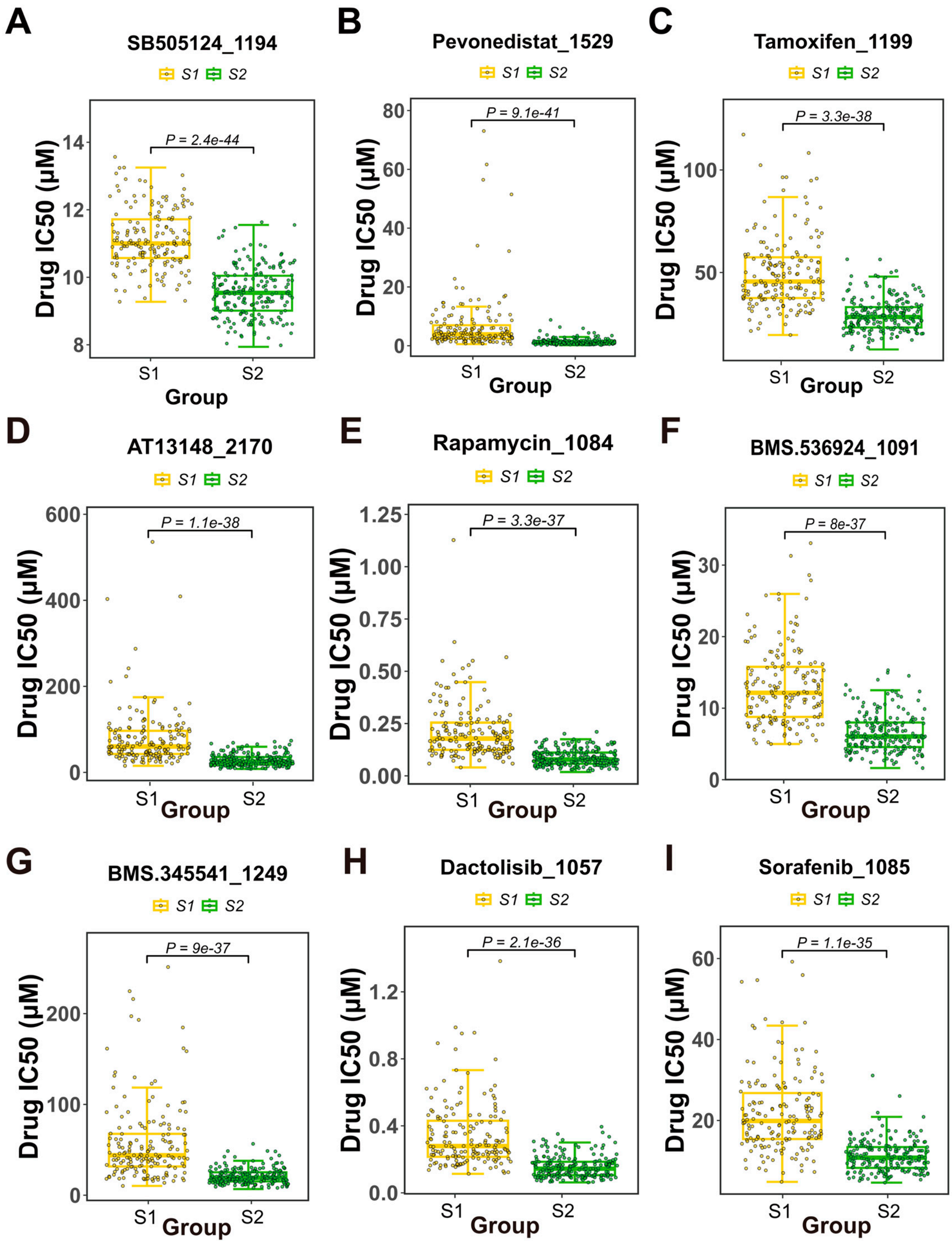

Figure S5. Sensitive drug in S2 subtype. (**A**–**I**) are SB505124, Pevonedistat, Tamoxifen, AT13148, Rapamycin, BMS.536924, BMS.345541, Dactolisib, and Sorafenib, respectively. *p*-values were determined via Wilcoxon test.

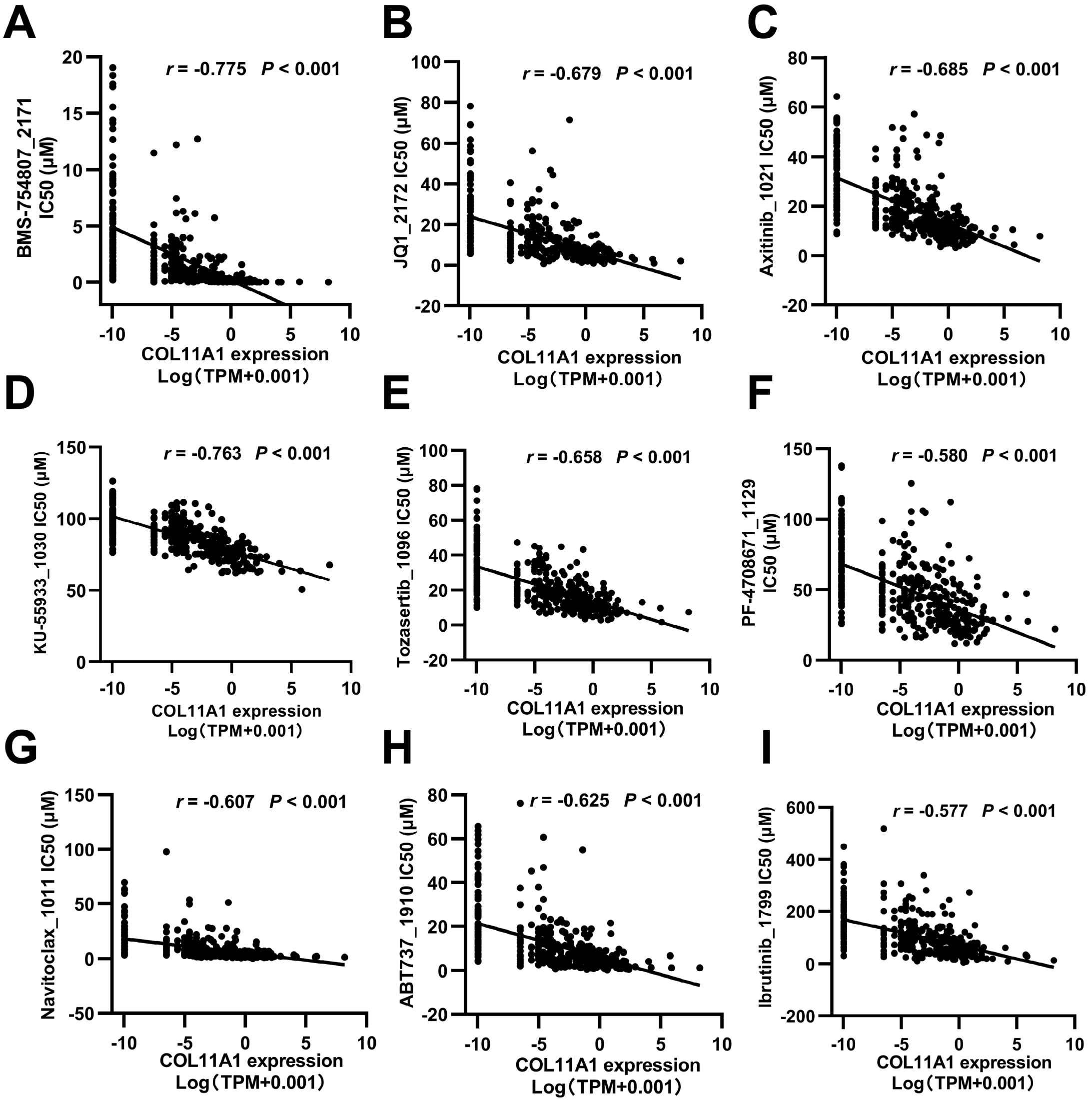

Figure S6. Correlation between IC50 of sensitive drugs in S1 subtype and COL11A1. (**A**–**I**) are BMS.754807, JQ1, Axitinib, KU.55933, Tozasertib, PF.4708671, Navitoclax, ABT737, and Ibrutinib, respectively. Correlation analyses were performed via Spearman analysis.

Table S1. Identification of characteristic genes via LASSO regression.

PLAUR	XXYLT1	INTS4	TMEM87B	EEF1E1	KCNK3	CENPH	ZNF879	ARTN	CAPRIN1
B3GALNT1	RHNO1	EPPIN	RASGEF1C	GATA3	ANK1	RALA	HPCAL4	SCG3	LRRN2
OR5D14	GABRG1	SUV39H2	GPR160	SOX4	F3	MYL2	CNGB1	ZC2HC1A	ITIH6
CC2D2B	SLC17A8	BMPR1B	RCVRN	SLC52A1	PLA2G4F	DDR1	NR0B1	OLAH	UBXN2A
BMP8A	ADCY2	CADPS	PSAPL1	CELF4	LRRN1	STX3	IGSF5	RHOV	SLC28A3
CLDN20	SLC6A17	RBM11	GPD1L	KRTAP20	HSF5	ZFP57	CPEB1	USH1G	C15orf39
TDRD5	MYH15	GAP43	DAND5	CCR10	TRIM17	GDF10	OTUD6A	FANCB	RPEL1
PLCB1	KLK9	TMEM145	ADARB2	GCSAML	PCDHA4	TYR	LRRC15	RIIAD1	MYH4
PRSS22	CDX1	ZNF492	SOSTDC1	CHRDL1	ACTL8	KRTAP19	COL11A1	NCCRP1	IL23R
TSPAN3	NUTM2G	UBE2U	GGT6	HOXC5	MISP				

Disclaimer/Publisher's Note: The statements, opinions and data contained in all publications are solely those of the individual author(s) and contributor(s) and not of MDPI and/or the editor(s). MDPI and/or the editor(s) disclaim responsibility for any injury to people or property resulting from any ideas, methods, instructions or products referred to in the content.
